# Effects of Therapeutic Horseback-Riding Program on Social and Communication Skills in Children with Autism Spectrum Disorder: A Systematic Review and Meta-Analysis

**DOI:** 10.3390/ijerph192114449

**Published:** 2022-11-04

**Authors:** Shihui Chen, Yanjie Zhang, Mengxian Zhao, Xiru Du, Yongtai Wang, Xiaolei Liu

**Affiliations:** 1 Department of Kinesiology, Texas A&M University, Texarkana, TX 75503, USA; 2 Physical Education Unit, School of Humanities and Social Science, Chinese University of Hong Kong, Shenzhen 518172, China; 3 School of Physical Education, Shenzhen University, Shenzhen 518060, China; 4 College of Sport Arts, Guangzhou Sport University, Guangzhou 510075, China; 5 College of Health Sciences and Technology, Rochester Institute of Technology, Rochester, NY 14623, USA; 6 Chinese Traditional Regimen Exercise Intervention Research Center, Beijing Sport University, Beijing 100084, China

**Keywords:** animal-assisted therapy, children, ASD, meta-analysis, social behavior, communication

## Abstract

Animal-assisted therapy has become a fast-growing and effective approach for remediating core impairments of children with ASD; however, recent systematic review studies on the effects of AAT in children with ASD have some limitations, including referral to a variety of animal-assisted interventions rather than to horseback-riding therapy alone and the absence of any meta-analysis in systematic reviews. A complete systematic review of the studies that describe the use of THR as an intervention is needed to specifically target the core impairments of children with ASD. The purpose of this study was to employ the systematic review method to synthesize research findings regarding the effects of THR programs on the social interaction and communication skills of children with ASD. We conducted a structured search in accordance with the Preferred Reporting Items for Systematic Reviews and Meta-Analyses (PRISMA) guidelines. We searched for potentially relevant studies in five databases (Web of Science, PubMed, CINAHL, Scopus, and SPORTDiscus) from inception until February 2022. In addition, we manually searched the bibliographies of the included studies to find articles that might otherwise have been missed. We considered articles eligible or ineligible if they satisfied specific inclusion or exclusion criteria. Our results showed that the THR program is an effective direct and alternative therapeutic program that can considerably improve the social behaviors and communication skills of children with ASD and can effectively impact autistic impairments in areas such as social awareness, social cognition, social motivation, and social communication. These findings are in line with those of previous studies; however, we did not find statistical evidence of any effect of THR on the autistic behaviors of irritability, stereotypy, and inappropriate speech. In conclusion, the findings produced by this meta-analysis study provide evidence that THR programs can considerably improve the social behaviors and communication skills of children with ASD.

## 1. Introduction 

According to a 2018 CDC report by the Johns Hopkins School of Public Health, the number of individuals with autism spectrum disorder (ASD) has dramatically increased in recent years; it has nearly tripled, from 0.67% to 1.85% compared with the figure for 2000 [1,2] (Johns Hopkins report, 2020; CDC 2018 report). ASD typically impairs interpersonal social interaction and communication, and results in stereotypic behaviors. In children, these autistic behaviors seriously affect their social life with their families and peers of a similar age. Physical activity, as an essential element for the development of children with ASD, has been extensively studied [3,4]. Participation in any animal-assisted physical activity, including horseback riding, allows children with ASD to enjoy a fun activity with their peers and to develop important interpersonal and social skills [5,6]. In addition, these physical activities can lead to improvements in self-esteem, behavior, and happiness in children with and without ASD [7,8]. As such, various physical activities in addition to horse-assisted therapeutic intervention can promote social interaction and communications and decrease levels of stress, anxiety, and inappropriate behavior in individuals with ASD [9,10,11,12].

The effects of animal-assisted therapy (AAT) as a therapeutic intervention and treatment for various chronic diseases and disabilities have been globally studied [13,14,15]. AAT was formally introduced by a psychiatrist who observed the interactions between a child with autism and a dog and proposed a new therapeutic practice based on the idea that interactions with dogs can improve the social communication skills of children with autism [16,17,18]. Since then, many medical professionals and therapists have used AAT to improve autistic children’s engagement in physical activity, release psychological stress and anxiety, and establish and promote a social and communication environment by means of interaction with various animals (e.g., dog, horse, or dolphin) [6]. AAT has also been used for individuals with depression, dementia, and pervasive developmental disorders [19]. A number of programs have been developed that provide excellent opportunities for social interaction, communication, and relaxation, with consequent reductions in perceived loneliness and psychological emotional stress [20,21,22,23]. Researchers have focused on the sensory impairment of children with cerebral palsy and have reported an increased range of motion and improved pain management [24].

Therapeutic horseback riding (THR), as an effective AAT, was originally used by an individual with polio as one of several equine-assisted therapeutic approaches and training regimes [12], and soon became popular, spreading from Europe to North America. Since then, the effects of THR programs have been investigated through various experimental studies, and their results have demonstrated the effectiveness of THR as an alternative treatment for various individuals with different disabilities [25,26]. For example, classic dressage has been used as an effective treatment technique for individuals with brain dysfunction [27]. A pilot study conducted by Gabriels et al. has shown that 10 weeks of THR could contribute to improving self-regulation behaviors, adaptive skills, and motor skills in children and adolescents with ASD [28]. Moreover, Lanning et al. investigated the behavior changes of children with ASD who received 6 weeks of THR and found significant improvements in children’ physical, mental, and social function [29]. The effectiveness of THR as an alternative treatment has continued to be investigated by many researchers [6,30,31].

Over the past few decades, the THR program used as an alternative medical treatment for people with physical and psychological disabilities has moved beyond the research setting and into practical therapeutic environments. Many medical professionals and physical therapists have designed and integrated different therapeutic recreational programs into their treatment to promote physical, social, and emotional well-being in their patients and reduce their stress and anxiety. Horse-related AAT offers companionship (interactions among instructor, horse, and patient) and provides an atmosphere of pleasure and relaxation, which may reduce the senses of isolation and anxiety [32,33].

After we completed a randomized control trial in which we used THR as a therapeutic activity to improve the social and communication skills of children with ASD [6], we found through a review of the literature that animal-assisted therapy has become a fast-growing and effective approach for remediating the core impairments of children with ASD. From the perspective of this study, we found that recent systematic review studies of the effects of AAT in children with ASD present some limitations, including reference to therapeutic interventions with animals other than horses (e.g., dog, cat, or dolphin) [34,35]. Some studies concerned different disabilities other than ASD (e.g., cerebral palsy, or intellectual disability) [36,37]; others focused on behavioral issues other than core impairments of social and communication stills. Some studies lacked a quality analysis of selected articles (i.e., effect size estimates) or offered a systematic review without meta-analysis [26,38]. Therefore, a complete systematic review is needed to identify those studies that use THR as an intervention that targeted the core impairments of children with ASD and used meta-analysis to select and analyze the articles of higher quality from different databases. Our purpose in this study was to employ the systematic review method to synthesize the research findings regarding the effects of THR programs on the social interaction and communication skills of children with ASD.

## 2. Materials and Methods

### 2.1. Search Strategy

We conducted a structured search in accordance with the Preferred Reporting Items for Systematic Reviews and Meta-Analyses (PRISMA) guidelines [39]. We searched for potential studies in five databases (Web of Science, PubMed, CINAHL, Scopus, and SPORTDiscus) from inception until February 2022. The key terms that we used were: (1) “animal assisted” OR “animal intervention” OR “animal therapy” OR “horseback riding” OR “equine-assisted intervention” OR “equine-assisted therapy”; AND (2) “autism spectrum disorder” OR “autism”; AND (3) “social interaction” OR “social function” OR “social communication”. In addition, we performed a manual search of the bibliographies of the included studies to find articles that might otherwise have been missed.

### 2.2. Inclusion and Exclusion Criteria 

We considered articles to have met inclusion criteria if they involved: (i) diagnosed children with autism; (ii) randomized controlled studies (RCTs); (iii) horse-assisted therapy in the intervention group; (iv) a usual-care or wait-list condition for the control group; (v) measurements of social function; (vi) publication in the English language. 

Exclusion criteria were: (i) the use of animals other than horses for trials; (ii) insufficient information for calculating the effect size (ES); (iii) case studies, observational studies, or review articles; (iv) duplicated study.

### 2.3. Data Extraction and Quality Assessment

We extracted detailed information concerning the author and publication year, characteristics of the study population such as sample size and ages of those involved, intervention protocols, diagnostic criteria, assessment tools, and outcomes. 

The quality of each study was assessed by two independent authors using the Physiotherapy Evidence Database (PEDro) scale [40]. This assessment scale has 11 domains: eligibility criteria, random allocation, concealed allocation, similar measures between groups at baseline, instructor blinding, assessor blinding, participant blinding, more than 85% dropout rate, intention-to-treat analysis, statistical comparison between groups, and ≥1 key outcome estimated. Each item is scored as 0 (absent) or 1 (present). The total score ranges from 0 to 10 points. Study quality is classified as excellent (9–10 points), good (6–8 points), fair (4–5 points), or poor (<4 points) based on the assessment. 

### 2.4. Statistical Analysis

We conducted a meta-analysis to measure the aggregative effect size (ES) of the effect of horse-assisted therapy on social function. In the analysis, we used standard mean differences (SMDs) to express the ESs by calculating the mean change from baseline to post intervention for the intervention and control groups. To analyze variables across studies, we used a random effects model with 95% confidence intervals (CIs) in the overall ESs calculated. We categorized the ESs as small (0.2–0.49), moderate (0.50–0.79), or large (≥0.8) based on the Higgins recommendation [41]. We used the *I*^2^ test to assess study heterogeneity, and the three cut-off points for the levels of low, moderate, and high heterogeneity were *I*^2^ = 25%, *I*^2^ = 50%, and *I*^2^ = 75%, respectively [42]. We conducted all statistical analyses using the Comprehensive Meta-Analysis program (version 2.2). We used a significance level of *p* < 0.05 in all analyses. 

## 3. Result

### 3.1. Search Results

**Figure 1:**
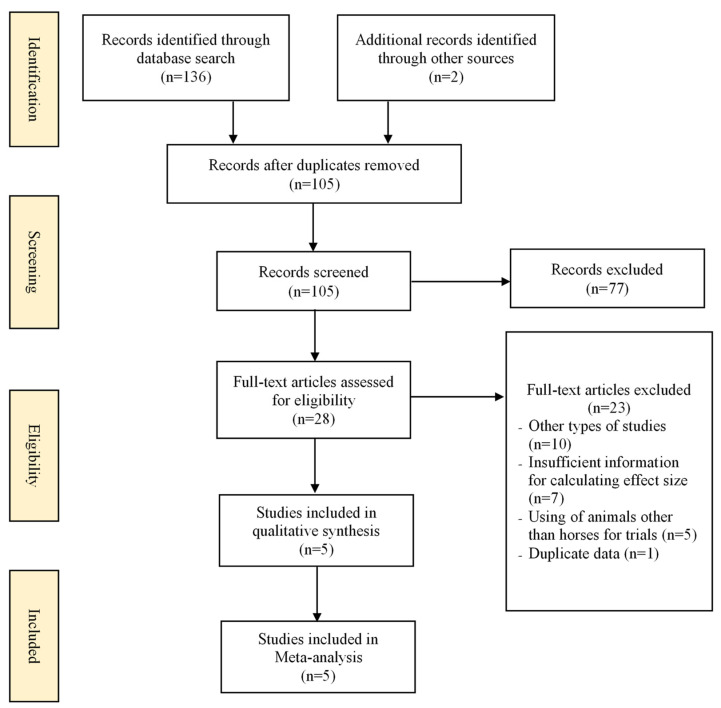
The flowchart of the study selection process.

The flowchart of selection studies is displayed in Figure 1. Initially, we identified 136 potentially suitable studies from the database and 2 studies from manual retrieval. Of these, we excluded 33 because of duplication, so we selected 105 full-text studies for a final review of titles and abstracts. We then reviewed 28 of these studies for eligibility by a reading of their full texts. Finally, we selected 5 studies [31,43,44,45,46] for meta-analysis.

### 3.2. Characteristics of Included Studies 

Table 1 shows detailed characteristics of the five included studies. Their years of publication ranged from 2009 to 2019. A total of 240 children with autism were included, and their ages ranged from 6 to 16 years old. All children in the intervention groups were offered horse-based therapy, which involved 45–70 min sessions of horse-assisted riding, 1–2 times per week, for 7–24 weeks. Those in control groups were placed on a waiting list or participated in regular activities. The measured outcomes included assessments of social function (social communication, social cognition, social awareness, and social motivation) and aberrant behavior (irritability, lethargy/social withdrawal, stereotypy, hyperactivity, and inappropriate speech). 

Our appraisal of study quality is presented in Table 2. We identified four good-quality studies (RCTs) and one fair-quality study (n-RCT) among the included studies. All studies used concealed allocation, subject blinding, and therapist blinding. One study used assessor blinding. The remaining items (similarity in key measures at baseline, and comparison of more than one outcome) were described in each study.

### 3.3. Meta-Analysis Results of Social Function 

We assessed the severity of symptoms associated with autism spectrum disorders (ASDs) using the SRS. The pooled meta-analysis results from the selected studies showed that horse-assisted therapy had a significant effect on the social functioning of children with ASD compared with the control group (Table 3). 

**Figure 2:**
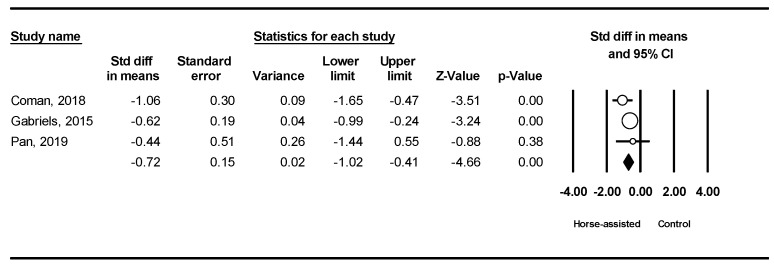
Forest plot showing the effects of horse-assisted therapy vs. control on social communication [43,44,46].

Among the five studies analyzed, the pooled meta-analysis results from the three studies in Figure 2 revealed that horse-assisted therapy significantly improved social communication in children with autism (SMD = −0.72 95% CI [−1.02, −0.41], *p* < 0.001).

**Figure 3:**
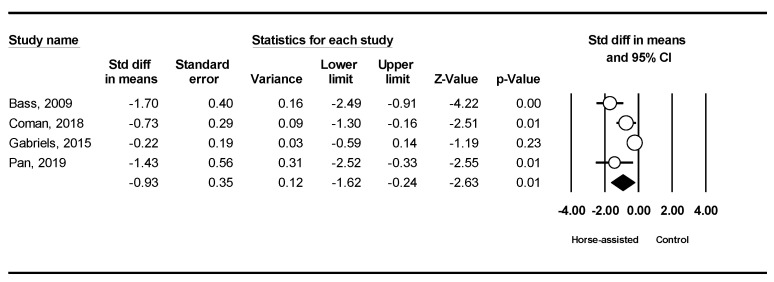
Forest plot showing the effects of horse-assisted therapy vs. control on social awareness [31,43,44,46].

The pooled meta-analysis results from the four studies in Figure 3 showed that horse-assisted therapy significantly improved social awareness in children with autism (SMD = −0.93, 95% CI [−1.62, −0.24], *p* < 0.001).

**Figure 4:**
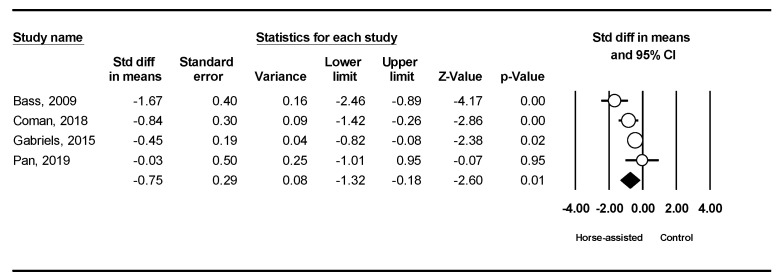
Forest plot showing the effects of horse-assisted therapy vs. control on social cognition [31,43,44,46].

The pooled meta-analysis results from the four studies in Figure 4 showed that horse-assisted therapy significantly improved social cognition in children with autism (SMD = −0.75, 95% CI [−1.32, −0.18], *p* < 0.001).

**Figure 5:**
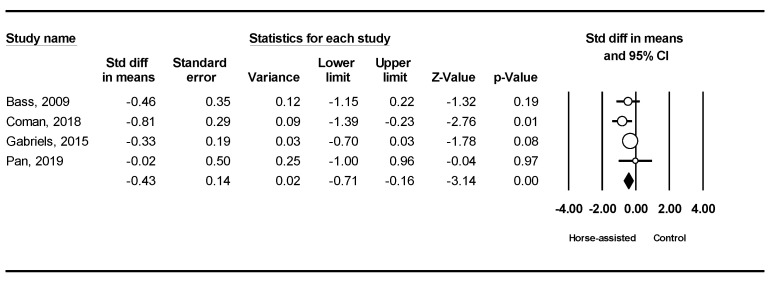
Forest plot showing the effects of horse-assisted therapy vs. control on social motivation [31,43,44,46].

The pooled meta-analysis results from the four studies in Figure 5 showed that horse-assisted therapy significantly improved social motivation in children with autism (SMD = −0.43, 95% CI [−0.71, −0.16], *p* < 0.001).

### 3.4. Meta-Analysis Results for Maladaptive Behaviors

The ABC-C scale was used to assess five maladaptive behavior problems: irritability, lethargy, hypersensitivity, stereotypy, and inappropriate speech. 

**Figure 6:**
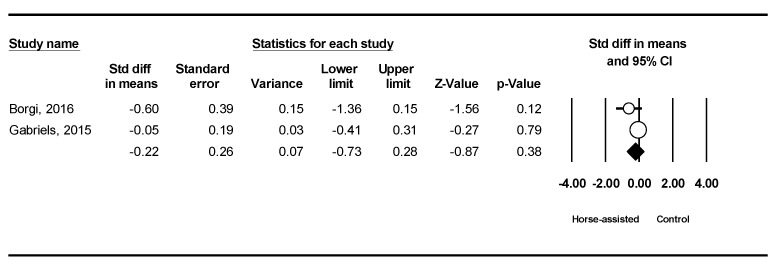
Forest plot showing the effects of horse-assisted therapy vs. control on irritability [12,44].

**Figure 7:**
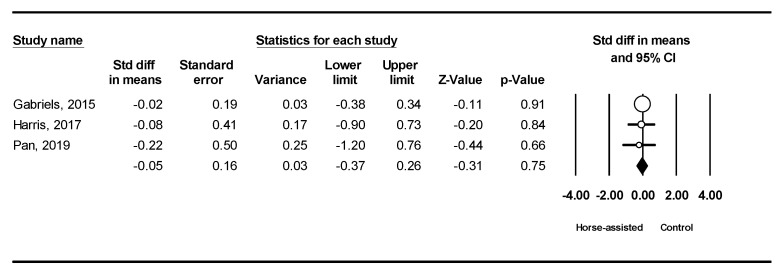
Forest plot showing the effects of horse-assisted therapy vs. control on stereotypy [44,45,46].

Among the five studies included, the pooled meta-analysis results from the two studies (in Figure 6) and three studies (in Figure 7) showed that horse-assisted therapy did not significantly improve irritability (SMD = −0.22, 95% CI [−0.73, 0.28], *p* = 0.38) or stereotypy behaviors in children with ASD (SMD = −0.05, 95% CI [−0.37, 0.26], *p* = 0.75), respectively.

**Figure 8:**
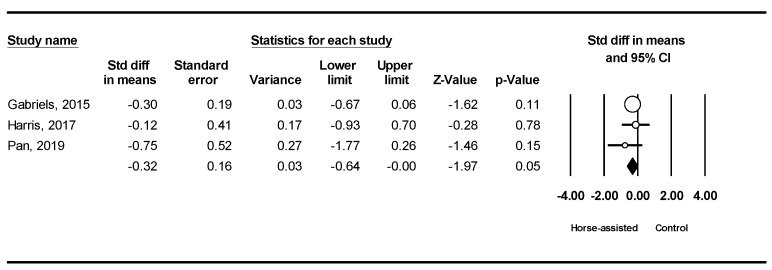
Forest plot showing the effects of horse-assisted therapy vs. control on lethargy [44,45,46].

Among the five studies included, the pooled meta-analysis results from the three studies in Figure 8 showed that horse-assisted therapy produced a significantly positive effect on lethargy in children with ASD (SMD = −0.32, 95% CI [−0.64, −0.001], *p* = 0.049).

**Figure 9:**
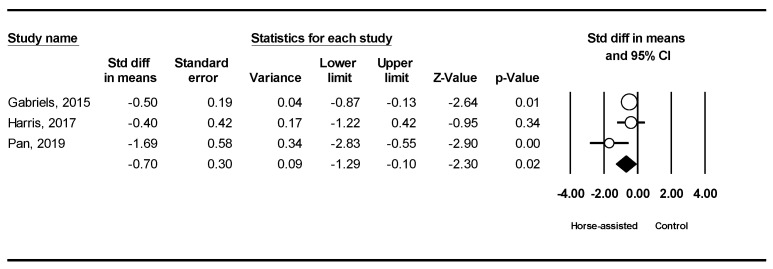
Forest plot showing the effects of horse-assisted therapy vs. control on hypersensitivity [44,45,46].

The pooled meta-analysis results from the three studies in Figure 9 showed that horse-assisted therapy produced a significantly positive effect on hypersensitivity in children with ASD (SMD = −0.70, 95% CI [−1.29, −0.10], *p* = 0.02).

**Figure 10:**
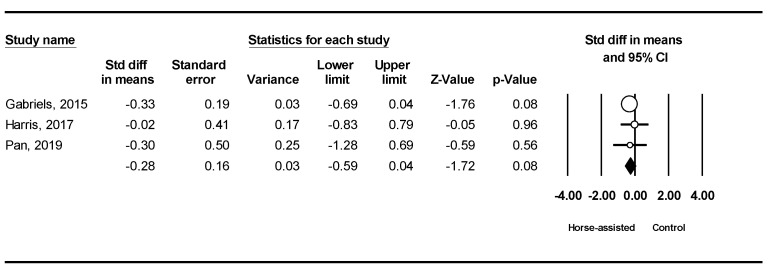
Forest plot showing the effects of horse-assisted therapy vs. control on inappropriate speech [44,45,46].

The pooled meta-analysis results from the three studies in Figure 10 showed that horse-assisted therapy did not produce a significant improvement in inappropriate speech behavior in children with ASD (SMD = −0.28, 95% CI [−0.59, 0.04], *p* = 0.08).

## 4. Discussion 

Our purpose in this study was to use the techniques of a systematic review and meta-analysis to synthesize the findings of previous studies regarding the effects of THR programs on the social interaction and communication skills of children with ASD. We selected 4 good-quality studies and 1 fair-quality study from a total of 138 initially identified AAI articles. A total of 240 participants were included and analyzed for this study. The results of the meta-analysis provided evidence that THR programs can considerably improve the social interaction and communication skills of children with ASD. Although we included only five articles in this study, they are most good quality and provide scientific evidence for our recommendation based on our quality assessment (see Table 2). All of the articles in our analysis described the use of horse-assisted therapy in their intervention groups focused on core ASD social and communication behaviors. We did not include studies of interventions involving the assistance of other animals (e.g., dog, cat, or dolphin) in our analysis, nor other study types, such as case studies, observational studies, or review articles. We calculated the ES of the studies, and we excluded studies with insufficient information for calculating the ES and those with low ES. By applying the above inclusion/exclusion criteria, we ensured that the quality of our study was well-controlled and focused only on THR interventions in relation to the social and communication skills of children with ASD. 

The results of the present meta-analysis demonstrate the considerable effects of THR programs on core behaviors of children with ASD, in line the findings of current AAT-related studies, which showed how animal assistance can promote social interaction and communication skills [11,12,38]. Our results also reveal effects upon social behaviors, as evaluated through the Social Responsiveness Scale (SRS), which includes subscales for social communication, social awareness, social cognition, and social motivation. All of these showed significant improvements overall compared with the waiting list control group. We think that these improvements in core behaviors of ASD are because well-organized THR programs provide opportunities for children with ASD to interact and communicate with horses, same-age peers, and instructors. In the five studies included, researchers provided evidence that improvements in social interaction and communication skills occurred because of the opportunities and stimulations gained from both verbal and nonverbal interactions with horses, peers, and trainers. Such interactions help children with ASD to better understand others, which is the most important means by which they can improve their social and communication skills.

The pooled meta-analysis results from two and three studies showed that no significant improvement was found concerning irritability or stereotypy behaviors in children with ASD. Our analysis is that these two areas of impairments are typical core behaviors of individuals with ASD and require careful, long-term remediation. The presentation of irritability behavior is usually associated with many other behavioral and emotional factors, including anxiety and mood difficulties, and these might trigger irritability behaviors. Stereotypy behavior is another impairment that needs long-term rehabilitation for correction because irritability and stereotypy behaviors in ASD may be attributed to neurobiological mechanisms, including dysfunction of the serotonergic, dopaminergic, and GABA neurotransmitter systems [47,48]. More specially, the basal ganglia are prone to dysfunction of the neurotransmitter system, which has been documented to contribute to stereotypical behaviors in ASD [49,50]. However, the changes in neurotransmitter systems require longer periods of exercise intervention (≥36 weeks) based on an animal model study [51]. In the studies included in our analysis, the experimental period was around 10 weeks, and a 10-week experimental period might be too short to reveal meaningful changes in the irritability and stereotypy behaviors of children with ASD. Our findings suggest this as a reasonable explanation for why no significant influence on irritability and stereotypy behaviors was observed. 

THR programs are associated with the experience of happiness that comes from being in contact with nature and animals, and, as such, programs are normally designed to integrate many specific rehabilitation activities into the remediating objectives related to the children’s behavioral problems; we think this is why THR programs are effective, enjoyable, and attractive to children with ASD. Although children with ASD may continue to demonstrate autistic behaviors such as irritability and stereotypy after undergoing a THR program, they typically show considerable improvements in other behaviors, such as social communication, social awareness, social cognition, and social motivation. Overall, such changes in behavior can produce many positive influences on the core social impairment of children with ASD.

## 5. Conclusions and Limitations

In this study, we used the techniques of a systematic review and meta-analysis to analyze existing research findings regarding the effects of THR programs on the social interaction and communication skill of children with ASD. The findings generated from the selected studies provided us evidence used to conclude that THR programs can notably remediate autistic impairments in social communication, social awareness, social cognition, and social motivation. This is in line with the findings of previous studies. The nature of THR programs, involving experiences of happiness, and being in contact with nature and animals, can considerably decrease levels of specific maladaptive behaviors of children with ASD, such as lethargy, hypersensitivity, and inappropriate speech. However, the findings from this meta-analysis do not provide statistical evidence of any influence upon the autistic behaviors of irritability and stereotypy, and we think that the short experimental periods used might have been an influencing factor in this regard. We also recognize that the findings derived from the existing RCT studies might be affected by the limited studies included in the present study, even though we followed a statistical meta-analysis procedure to control the quality of the selected articles. In future studies, researchers may need to increase the number of studies used to prevent the possible statistical influences of the small number of studies.

## Figures and Tables

**Table 1:** Characteristics of randomized controlled trials in the meta-analysis [31,43,44,45,46].

Study/ Country	Participants		Intervention(s)		Diagnostic Criteria	Outcomes
	Sample Size	Age (Years) Range	Experiment Group	Control Group		
Bass et al. (2009) [31] USA	34 E = 19 C = 15	Me = 6.95 Mc = 7.73 (5–10 y)	70 min/week (including 10 min warm-up) 12 weeks Equine-assisted (EA) intervention	Wait-list	DSM-IV-TR ASD	Social Responsiveness Scale (social awareness, social cognition, social motivation)
Coman et al. (2018) [43] USA	50 E = 25 C = 25	8.7 (7–12 y)	70 min/week (including 10min warm-up) 12 weeks Equine-assisted (EA) intervention	Wait-list	DSM-IV-TR ASD	Social Responsiveness Scale (SRS) (social awareness, social cognition, social communication, social motivation)
Gabriels et al. (2015) [44] USA	116 E = 58 C = 58	10.2 (6–16 y)	45 min/week 10 weeks Therapeutic horseback riding	Barn Activity Control	Autism Diagnostic Observation Schedule (ADOS) or ADOS-second edition (ADOS-2)	Social Responsiveness Scale (SRS) (social awareness, social cognition, social communication, social motivation) Aberrant Behavior Checklist–Community (ABC-C) (Irritability, and Hyperactivity)
Harris (2017) [45] UK	24 M = 10 C = 14	7.38 6.08–9.33 y	2 × 45 min/week 7 weeks Horseback riding intervention	Wait-list	Clinician	Childhood Autism Rating Scale Aberrant Behavior Checklist-Community Edition (ABC-C) (irritability, lethargy/social withdrawal, stereotypy, hyperactivity, and inappropriate speech behaviors)
Pan et al. (2019) [46] USA	16 E = 8 C = 8	Me = 11.88 Mc = 9.80 (6-16 y)	45 min/week 10 weeks Therapeutic horseback riding	No horse interaction, barn activity	ADOS-2	Social Responsiveness Scale (SRS) (social awareness, social cognition, social communication, social motivation) Aberrant Behavior Checklist–Community (ABC-C) (irritability, lethargy/social withdrawal, stereotypy, hyperactivity, and inappropriate speech behaviors)

Note: y = years; Me = mean age in experimental group; Mc = mean age in control group; ADOS-2 = Autism Diagnostic Observation Schedule—2nd Edition.

**Table 2:** Methodological quality of the included studies [31,43,44,45,46].

Study	Score	Methodological Quality	PEDro Item Number										
			1	2	3	4	5	6	7	8	9	10	11
Bass et al. (2009) [31]	6	Good	1	1	0	1	0	0	0	1	1	1	1
Coman et al. (2018) [43]	7	Good	1	1	0	1	0	0	1	1	1	1	1
Gabriels et al. (2015) [44]	6	Good	1	1	0	1	0	0	0	1	1	1	1
Harris et al. (2017) [45]	5	Fair	1	0	0	1	0	0	0	1	1	1	1
Pan et al. (2019) [46]	6	Good	1	1	0	1	0	0	0	1	1	1	1
Studies were classified as excellent (9–10), good (6–8), fair (4–5), or poor (<4).													

Scale of item score: 0 = absent, 1 = present. PEDro scale criteria are (1) eligibility criteria; (2) random allocation; (3) concealed allocation; (4) similarity at baseline on key measures; (5) subject blinding; (6) therapist blinding; (7) assessor blinding; (8) more than 85% follow-up of at least one key outcome; (9) intention-to-treat analysis; (10) between-group statistical comparison for at least one key outcome; and (11) point estimates and measures of variability provided for at least one key outcome.

**Table 3:** Meta-analysis results for the effects of horse-assisted therapy vs. control intervention.

Outcome	Number of Trials	SMD	95% CI	*I^2^* %	Between-Group Homogeneity			Publication Bias
					*Q*-Value	df (*Q*)	*p*-Value	Egger’s Test (*p*)
Social communication	3	−0.72	−1.02 to −0.41	0%	1.87	2	0.39	0.93
Social awareness	4	−0.93	−1.62 to −0.24	78.5%	13.95	3	0.000	0.08
Social cognition	4	−0.75	−1.32 to −0.18	68.8%	9.62	3	0.02	0.64
Social motivation	4	−0.43	−0.71 to −0.16	0%	2.62	3	0.45	0.99
Irritability	2	−0.22	−0.73 to 0.28	40.0%	1.66	1	0.20	-
Stereotypy	3	−0.05	−0.37 to 0.26	0%	0.15	2	0.93	0.17
Lethargy/social withdrawal	3	−0.32	−0.64 to −0.001	0%	0.95	2	0.62	0.39
Hyperactivity	3	−0.70	−1.29 to −0.10	49.9%	4.00	2	0.14	0.52
Inappropriate speech	3	−0.28	−0.59 to 0.04	0%	0.46	2	0.80	0.83

## Data Availability

The data can be directed to the corresponding author.
